# High prevalence of metabolic diseases, liver steatosis and fibrosis among Chinese psychiatric patients

**DOI:** 10.1186/s12888-023-04684-1

**Published:** 2023-03-28

**Authors:** Huixia Li, Chi Chen, Yi Chen, Bing Han, Yingchao Chen, Jing Cheng, Ningjian Wang, Bin Wang, Yingli Lu

**Affiliations:** Institute and Department of Endocrinology and Metabolism, Shanghai Ninth People’s Hospital, Shanghai JiaoTong University School of Medicine, Shanghai, 200011 China

**Keywords:** Psychiatric patients, Metabolic disease, Liver steatosis, Liver fibrosis

## Abstract

**Background:**

We aimed to investigate the differences of metabolic disorders between the general population and psychiatric patients, with an emphasis on the prevalence and influencing factors of liver fibrosis in psychiatric patients.

**Methods:**

A total of 734 psychiatric patients and 734 general population matched for age, sex, and BMI were enrolled from Shanghai, China. All participants underwent blood pressure, glucose, lipid profile measurements, and anthropometric parameters including body weight, height and waist circumference. FibroScan examinations were also performed on psychiatric patients. Liver steatosis and fibrosis were diagnosed by controlled attenuation parameter (CAP) and liver stiffness measurement (LSM) by professional staff.

**Results:**

Compared with the general population, psychiatric patients revealed significantly higher burden of metabolic disorders. The overall prevalence of liver steatosis (CAP ≥ 233 dB/m) and fibrosis (LSM ≥ 7.0 kPa) was 48.7% and 15.5% in psychiatric patients. Psychiatric patients with liver steatosis or fibrosis showed worse metabolic profile. Meanwhile, the prevalence of liver fibrosis was also significantly higher in patients with overweight, central obesity, diabetes, hypertension, metabolic syndrome, and liver steatosis. In logistic regression analyses, age, BMI and visceral adiposity index were independent risk factors for liver fibrosis in psychiatric patients. Additionally, antipsychotic medication was suggested to be associated with an increased risk of liver fibrosis in psychiatric patients with liver steatosis.

**Conclusions:**

Prevalence of liver steatosis and fibrosis is high in Chinese psychiatric patients. Those with antipsychotic polypharmacy and obesity are at high risk, and may benefit from early liver assessment in preventing fibrosis progression.

**Supplementary Information:**

The online version contains supplementary material available at 10.1186/s12888-023-04684-1.

## Introduction

The prevalence of mental disorders has increased considerably over the past four decades, affecting people across all regions of the world [1]. Among Chinese adults, the lifetime prevalence of any mental disorder was estimated to be 16.6%, imposing a huge burden on public health system [2]. Alarming data have shown that psychiatric patients die about 10–20 years earlier than their peers who are not suffering from mental illnesses [3–5]. Although the reason is multi-factorial [6], the high risk of metabolic diseases accounts for the excess mortality in people with mental disorders[7]. To make matters worse, patients with severe mental illness (SMI) who have metabolic diseases are less likely to be diagnosed and receive treatment [8]. Previous studies have shown that people with mental illness have a higher prevalence of metabolic disorders [9–11]. In a meta-analysis that included 198 studies and 52,678 participants, compared to the general population, people with SMI had significantly increased risk for abdominal obesity, low high-density lipoprotein (HDL), hypertriglyceridemia, and hyperglycemia [12]. In addition, 34.4% of patients with SMI met the criteria of metabolic syndrome and had a 58% higher risk compared to the general population. Nevertheless, most previous studies were conducted on the western population, the prevalence of metabolic disorders in Chinese psychiatric patients is relatively unexplored.

As a result of the growing obesity epidemic, nonalcoholic fatty liver disease (NAFLD) has become a major public health issue [13]. Due to the higher incidence of metabolic diseases in psychiatric patients, the prevalence of NAFLD may be higher in psychiatric patients compared with the general population. Liver-related morbidity and mortality in NAFLD patients are associated with the development of nonalcoholic steatohepatitis (NASH), which can progress to fibrosis and cirrhosis lesions [14]. The incidence of liver cirrhosis in NASH patients is as high as 15–25% within 10 to 15 years [15]. Hepatic fibrosis increases risk of liver-related complications, such as cirrhosis, liver failure, hepatocellular carcinoma (HCC) and death [16–18]. In addition, NAFLD is closely related with important extrahepatic manifestations, such as cardiovascular disease [CVD], chronic kidney disease [CKD], and certain extrahepatic cancers, that can further increase the disease burden, and CVD is the most common cause of death in NAFLD patients [16, 19].

Knowledge on the epidemiology of liver steatosis and fibrosis in psychiatric patients is relatively incomplete due to the limitations of various diagnostic modalities. Although liver biopsy is considered the gold standard, it is not feasible to apply to a large population. Abdominal ultrasonography is easily accessible but is a qualitative and subjective test which is operator dependent, and is insensitive in cases of mild liver steatosis [20]. In comparison, vibration-controlled transient elastography is quick and convenient to implement and has high patient acceptance [21]. It has high accuracy and reproducibility when used to assess advanced fibrosis and cirrhosis. With this non-invasive technique, it is now possible to detect liver steatosis and fibrosis simultaneously in a relatively large population.

In this study, we aimed to investigate the differences in metabolic disorders between the general population and psychiatric patients, and further investigate the factors that influence the prevalence of liver steatosis and fibrosis in Chinese psychiatric patients.

## Materials and methods

### Study design and participants

The population of this study consists of two groups, including the general population and the psychiatric patients. The inclusion criteria were as follows: age ≥ 18 years old; the integrity of personal information and inspection data; cooperating with the inspection. The exclusion criteria were as follows: severe heart, liver, and renal insufficiency; cerebrovascular accident; personal history of malignancy; acute febrile illness; pregnant women; inability to provide informed consent. The general population was from Shanghai area of the SPECT-China study [22–24]. They were without mental illnesses, who were not receiving any psychotropic medication or being treated for acute alcohol or drug intoxication. The psychiatric patients were from the Shanghai Huangpu and Pudong Mental Health Center. They were adult patients diagnosed by psychiatrists. In the study about the difference of metabolic disorders between the general people and psychiatric patients, the general population (n = 3505) were taken as the control group and the psychiatric patients (n = 743) as the case group, and the propensity score match (PSM) was applied so that there was no statistical difference in the distribution of gender, age and BMI between the two groups. In total,1468 participants were involved in the final analyses, including 734 general population and 734 psychiatric patients. In the study about risk factors that influence the prevalence of liver fibrosis, 669 psychiatric patients with valid LSM values were involved in the final analyses.

The study protocol was approved by the Ethics Committee of Shanghai Ninth People’s Hospital, Shanghai Jiao Tong University School of Medicine. All procedures were performed in accordance with the ethical guidelines of the 1975 Declaration of Helsinki and the ethical standards of the responsible institutional and national committee on human experimentation. We obtained written consent from all participants enrolled in the study.

### Data collection

The information on sociodemographic characteristics, medical history, family history, and lifestyle factors was accessed by the same group of trained and experienced personnel from the SPECT-China study and the METAL study [25–27] through using a detailed questionnaire. Anthropometric measurements including weight, height, neck circumference (NC), waist circumference (WC), hip circumference (HC) and blood pressure are taken by trained staff following the standard protocol described earlier [28]. Height and weight were measured with participants standing without shoes and in lightweight clothes to the nearest 0.1 cm and 0.1 kg. WC was measured on the midaxillary line between the lowest border of the rib cage and the top of the iliac crest to the nearest 0.1 cm. NC was measured below the cricoid cartilage and then at the level of the mid-cervical spine to the nearest 0.1 cm. HC was measured at the widest part of the hip at the level of the greater trochanter to the nearest 0.1 cm. Blood pressure was measured in the nondominant arm by an automated electronic device (HEM-752 FUZZY, Omron, China). After a 5-min rest, blood pressure measurements were repeated three times with 1-min intervals. The average systolic and diastolic blood pressures of the three readings were recorded on the questionnaire. Carotid plaque was diagnosed by ultrasound. Liver steatosis was diagnosed by ultrasound in the general population. BMI was calculated as weight in kilograms divided by squared height in meters. The LAP and VAI were calculated as follows [25]:

Males: VAI = WC (cm)/[39.68 + 1.88 × BMI (kg/m^2^)] × [TG (mmol/L)/1.03] × [1.31/HDL (mmol/L)]

LAP = [WC (cm)-65] × TG (mmol/L).

Females: VAI = WC (cm)/ [36.58 + 1.89 × BMI (kg/m2)] × [TG (mmol/L)/0.81] × [1.52/HDL (mmol/L)]

LAP = [WC (cm)-58] × TG (mmol/L).

### Biochemical measurements

Blood samples were drawn in the fasting state between 6:00 am and 9:00 am. The blood samples for the plasma glucose test were collected into vacuum tubes with the anticoagulant sodium fluoride and centrifuged within 2 h after collection. The serum was aliquoted and frozen at -20 °C after collection and then shipped by air within 2–4 h on dry ice to a central laboratory. Glycated hemoglobin (HbA1c) was assessed by high-performance liquid chromatography (MQ-2000PT, Medconn, China). Fasting plasma glucose (FPG), triglycerides (TG), total cholesterol (TC), HDL and low-density lipoprotein (LDL) were performed with a Beckman Coulter AU 680 (Brea, USA) [25].

### FibroScan examination

In psychiatric patients, liver stiffness measurement (LSM) and CAP were obtained using FibroScan handy (Echosens, Paris, France). All patients were fasted for at least 8 h before the FibroScan examination. The LSM score was represented by the median of 10 measurements, and it was considered reliable only if at least 10 successful acquisitions were obtained, with IQR-to-median ratio ≤ 0.3. The CAP score was represented by the median value. Because the meaning of IQR-to-median ratio for CAP is less well defined, CAP measurements were considered reliable if 10 successful acquisitions were obtained. All patients were first examined with the M probe to obtain both LSM and CAP. If the M probe failed, the XL probe suitable for obese patients was applied.

Liver steatosis was assessed by CAP, and the best CAP cutoff for S2 or greater disease was 233 dB/m [24, 29]. Liver fibrosis was assessed by LSM, and the best LSM cutoff for F2 or greater disease was 7.0 kPa [30].

### Definition of variables

Diabetes mellitus was defined as FPG ≥ 7.0mmol/L, or HbA1c ≥ 6.5%, or a self-reported previous diagnosis of Diabetes mellitus. Central obesity was defined as WC ≥ 90 cm for males, or WC ≥ 80 cm for females. Metabolic syndrome was defined according to International Diabetes Federation [31]:

Central obesity: WC ≥ 90 cm for males, or WC ≥ 80 cm for females.

Plus any two:

Raised triglycerides: >150 mg/dL (1.7 mmol/L) or specific treatment for this lipid abnormality.

Reduced HDL-cholesterol: <40 mg/dL (1.03 mmol/L) in men or < 50 mg/dL (1.29 mmol/L) in women or specific treatment for this lipid abnormality.

Raised blood pressure: systolic ≥ 130 mm Hg or diastolic ≥ 85 mm Hg or treatment of previously diagnosed hypertension.

Raised fasting plasma glucose: fasting plasma glucose ≥ 100 mg/dL (5.6 mmol/L) or previously diagnosed type 2 diabetes.

If above 5.6 mmol/L or 100 mg/dL, oral glucose tolerance test is strongly recommended, but is not necessary to define presence of syndrome.

### Statistical analysis

Data analyses were performed with IBM SPSS Statistics, version 26 (IBM Corporation, Armonk, NY, USA). *P* value (two-sided) < 0.05 indicated significance. Continuous variables were expressed as the mean ± standard deviation (SD) or the median with an interquartile range (25%, 75%), and categorical variables were presented as percentages (%). The Student’s t test or Mann–Whitney U test was used for continuous variables, and the Chi-square test was used for dichotomous variables. Binary Logistic regression analysis was performed to analyze the independent risk factors for liver fibrosis in psychiatric patients.

## Results

### Comparison of general population and psychiatric patients

As shown in Table 1, the education level of general population was significantly higher than psychiatric patients (P < 0.05). Psychiatric patients have significantly higher prevalence of liver steatosis, hyperlipidemia, diabetes mellitus, central obesity, metabolic syndrome than general population (all P < 0.05). Compared with the general population, LAP, VAI, WC, NC were significantly higher, and HDL was significantly lower in psychiatric patients (all P < 0.05). On the contrary, HC, SBP, DBP, TG, TC, LDL, FPG, HbA1c were all significantly higher in general population than in psychiatric patients (all P < 0.05). However, no differences in the prevalence of hypertension were found between the two groups (P > 0.05).

**Table 1:** Comparison of glycolipid metabolism in general population and psychopaths

	Total (person)	General population	Psychiatric patients	p value
High school or above	1332	45.4%	39.0%	0.019
Liver steatosis	1301	48.4%	58.4%	0.000
Hypertension	1468	52.5%	55.9%	0.190
Hyperlipemia	1468	47.5%	69.3%	0.000
Diabetes mellitus	1468	18.8%	32.8%	0.000
Central obesity	1468	37.7%	63.9%	0.000
Metabolic syndrome	1468	27.5%	40.7%	0.000
LAP	1468	26.4(15 ~ 45.9)	33.2(19.7 ~ 57.3)	0.000
VAI	1468	1.45(0.94 ~ 2.42)	1.85(1.2 ~ 2.87)	0.000
WC (cm)	1468	82.5(76 ~ 89.6)	89(82 ~ 96)	0.000
NC (cm)	1468	34.5(32 ~ 37)	35.2(33 ~ 38)	0.000
HC (cm)	1468	95(90 ~ 99)	93.8(89 ~ 98)	0.000
SBP (mm Hg)	1468	132(119 ~ 147)	129(116 ~ 146)	0.033
DBP (mm Hg)	1468	78(70.8 ~ 86)	76(68 ~ 86)	0.014
TG (mmol/L)	1468	1.3(0.95 ~ 1.86)	1.24(0.87 ~ 1.77)	0.009
HDL (mmol/L)	1468	1.34(1.12 ~ 1.54)	1.09(0.92 ~ 1.27)	0.000
TC (mmol/L)	1468	5.18(4.58 ~ 5.89)	4.41(3.87 ~ 5.12)	0.000
LDL (mmol/L)	1468	3.22(2.72 ~ 3.82)	2.81(2.4 ~ 3.31)	0.000
FPG (mmol/L)	1468	5.43(5.05 ~ 6.08)	5.4(4.9 ~ 6.1)	0.016
HbA1c (%)	1468	5.6(5.2 ~ 6)	5.4(5.1 ~ 5.8)	0.000

Continuous variables were expressed as the mean ± standard deviation (SD) or the median with an interquartile range (25%, 75%), and categorical variables were presented as percentages (%)

FLD fatty liver disease, US ultrasound, LAP lipid accumulation product, VAI visceral adiposity index, WC waist circumference, NC neck circumference, HC hip circumference, SBP systolic blood pressure, DBP diastolic blood pressure, TG triglycerides, HDL high-density lipoprotein, TC total cholesterol, LDL low-density lipoprotein, FPG fasting plasma glucose, HbA1c glycated hemoglobin

### The characteristics of psychiatric patients

#### Medication use of psychiatric patients

**Fig. 1:**
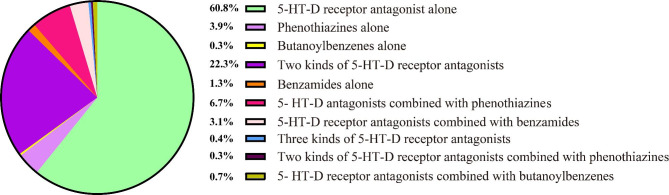
Current situation of antipsychotic medication use in psychiatric patients

We described the use of medication in 669 psychiatric patients, 85.2% of whom were schizophrenic. As shown in Fig. 1, Most patients (60.8%) received 5-hydroxytryptamine-dopamine (5-HT-D) receptor antagonists alone, followed by phenothiazines (3.9%) and benzamides (1.3%), and nearly one in five patients (22.3%) received two kinds of 5-HT-D receptor antagonists. Therefore, 5-HT-D receptor antagonists were the main drugs in patients with schizophrenia.

#### Demographic characteristics of psychiatric patients

Supplementary Table 1 illustrated that the differences of metabolic disorders between the two groups stratified according to sex (total 669 persons), educational status (total 543 persons) and age (total 669 persons), respectively. In general, women had a worse metabolic profile than men. And the disorders of glucose and lipid metabolism were more severe in older age.

#### Comparison of glycolipid metabolism in psychiatric patients

Table 2 illustrated that subjects with liver steatosis have significantly higher prevalence of hypertension, hyperlipemia, diabetes mellitus, central obesity, metabolic syndrome than their counterparts without liver steatosis (all P < 0.05). Compared with subjects without liver steatosis, BMI, WC, NC, HC, SBP, DBP, TG, TC, LDL, LAP, VAI, FPG, HbA1c were significantly higher, and HDL was significantly lower in subjects with liver steatosis (all P < 0.05). No differences in the prevalence of carotid plaque and age were found between the two groups (both P > 0.05). Meanwhile, subjects with liver fibrosis have significantly higher prevalence of carotid plaque, liver steatosis, hypertension, diabetes mellitus, dyslipidemia, central obesity, metabolic syndrome than their peers without liver fibrosis (all P < 0.05). Compared with subjects without liver fibrosis, age, BMI, WC, NC, HC, SBP, TG, LAP, VAI, FPG, HbA1c were significantly higher, and HDL was significantly lower in subjects with liver fibrosis (all P < 0.05). No differences in the prevalence of DBP, TC, and LDL were found between the two groups (all P > 0.05).

**Table 2:** Comparison of glycolipid metabolism in psychiatric patients(n = 669)

	Subjects without liver steatosis	Subjects with liver steatosis	p value	Subjects without liver fibrosis	Subjects with liver fibrosis	p value
Drinking	5.0%	4.6%	0.830	5.0%	3.8%	0.804
Carotid plaque	47.5%	46.9%	0.879	45.3%	57.7%	0.020
Liver steatosis	…	…	…	45.7%	65.4%	0.000
Hypertension	44.9%	66.6%	0.000	52.4%	72.1%	0.000
Hyperlipemia	58.6%	79.4%	0.000	66.5%	80.8%	0.004
Diabetes mellitus	26.5%	38.3%	0.001	30.1%	44.2%	0.005
Central obesity	45.2%	81.9%	0.000	60.2%	78.8%	0.000
Metabolic syndrome	32.9%	74.2%	0.000	49.6%	72.1%	0.000
Age (years)	65(58 ~ 70)	63.5(58 ~ 69)	0.170	64(57 ~ 69.5)	67(59.5 ~ 71)	0.012
BMI (kg/m²)	21.9(19.8 ~ 24.1)	25.7(23.6 ~ 27.9)	0.000	23.5 ± 3.6	26.3 ± 4.1	0.000
WC (cm)	85(78 ~ 90)	94(87.8 ~ 100)	0.000	88.2 ± 9.9	95.2 ± 11.6	0.000
NC (cm)	34.5(32 ~ 36.5)	37(34 ~ 39.1)	0.000	35(32.5 ~ 37.5)	37(34 ~ 40)	0.000
HC (cm)	90(87 ~ 95)	96(92 ~ 100.5)	0.000	92.5(88 ~ 98)	96.5(92 ~ 101.9)	0.000
SBP (mm Hg)	124(110 ~ 139)	135.5(122 ~ 150.3)	0.000	129(114 ~ 145)	138(123.3 ~ 153.5)	0.000
DBP (mm Hg)	73(66 ~ 83)	79(71 ~ 88.3)	0.000	76(68 ~ 86)	77.5(68.3 ~ 86)	0.444
TG (mmol/L)	1.06(0.79 ~ 1.38)	1.57(1.11 ~ 2.17)	0.000	1.22(0.85 ~ 1.71)	1.49(1.08 ~ 1.96)	0.001
HDL (mmol/L)	1.12(0.96 ~ 1.33)	1.03(0.88 ~ 1.18)	0.000	1.09(0.93 ~ 1.28)	1.01(0.87 ~ 1.18)	0.001
TC (mmol/L)	4.37 ± 0.91	4.64 ± 1.00	0.000	4.41(3.85 ~ 5.11)	4.48(3.92 ~ 5.12)	0.842
LDL (mmol/L)	2.74 ± 0.66	2.97 ± 0.72	0.000	2.81(2.39 ~ 3.31)	2.89(2.4 ~ 3.29)	0.597
LAP	22.8(13.8 ~ 36.7)	51.2(31.6 ~ 75.6)	0.000	31.6(17.8 ~ 53.3)	52.4(31 ~ 79)	0.000
VAI	1.48(1 ~ 2.13)	2.54(1.62 ~ 3.74)	0.000	1.8(1.16 ~ 2.71)	2.53(1.6 ~ 3.43)	0.000
HbA1c (%)	5.3(5 ~ 5.7)	5.5(5.1 ~ 6.1)	0.000	5.4(5.1 ~ 5.8)	5.6(5.2 ~ 6.2)	0.000
FPG (mmol/L)	5.3(4.83 ~ 5.9)	5.5(4.99 ~ 6.23)	0.008	5.4(4.9 ~ 6.1)	5.7(5.1 ~ 6.48)	0.003

Continuous variables were expressed as the mean ± standard deviation (SD) or the median with an interquartile range (25%, 75%), and categorical variables were presented as percentages (%)

LAP lipid accumulation product, VAI visceral adiposity index, BMI body mass index, WC waist circumference, NC neck circumference, HC hip circumference, SBP systolic blood pressure, DBP diastolic blood pressure, TG triglycerides, HDL high-density lipoprotein, TC total cholesterol, LDL low-density lipoprotein, FPG fasting plasma glucose, HbA1c glycated hemoglobin

#### Prevalence of liver fibrosis by different groups in psychiatric patients

**Fig. 2:**
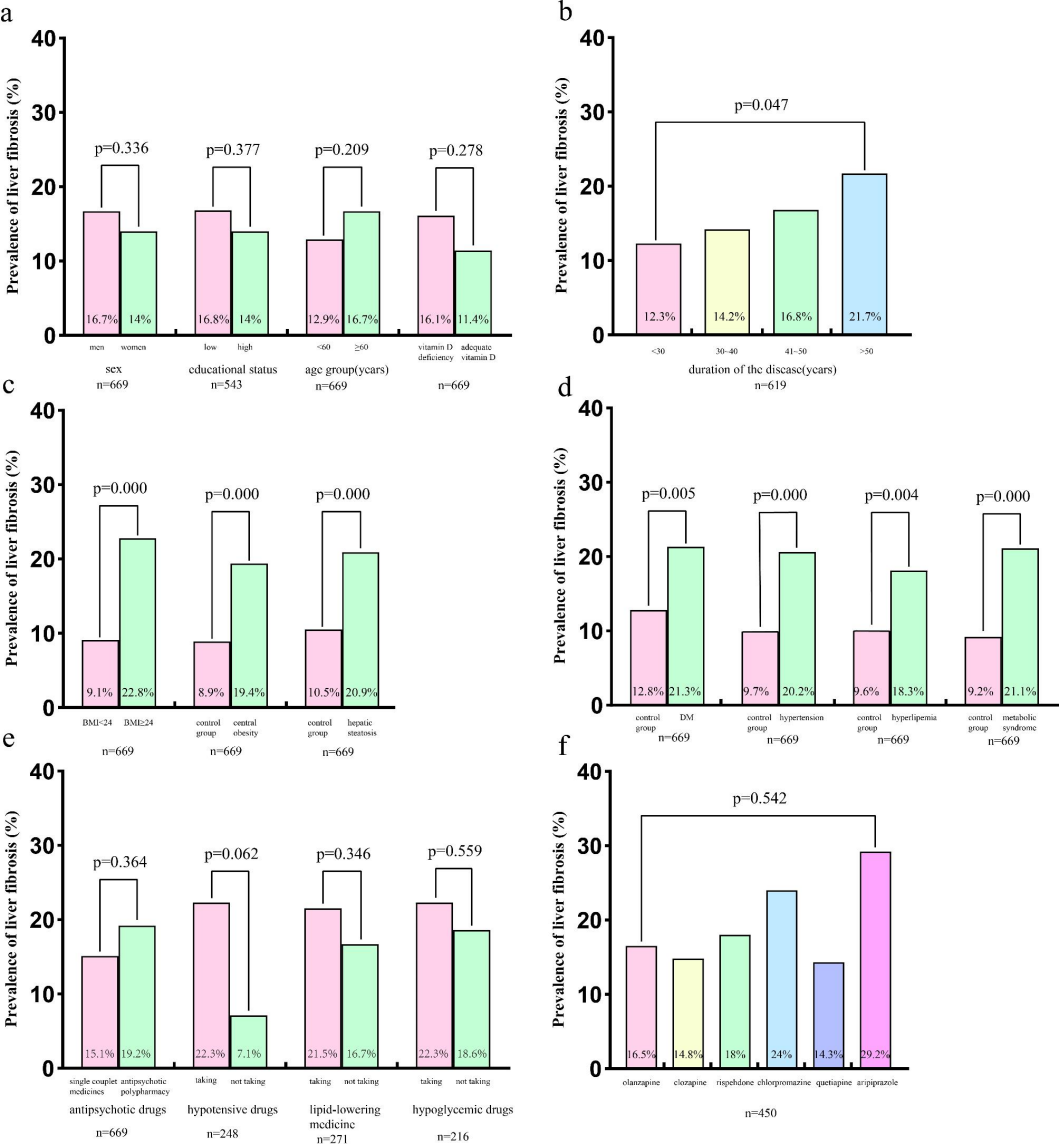
Prevalence of liver fibrosis according to demographic and metabolic factors

Fig. 2a indicated that the prevalence of liver fibrosis was higher in men than in women, in low educational status than in high educational status, in patients aged 60 or older than in their younger peers, in patients with vitamin D deficiency than in those with adequate vitamin D, although the differences were not statistically significant (P > 0.05). Figure 2b showed that the prevalence of liver fibrosis increased gradually with duration of disease (P < 0.05). Figure 2c indicated that the prevalence of liver fibrosis was significantly higher in patients with overweight (BMI ≥ 24), central obesity, and liver steatosis, respectively (all P < 0.05). Figure 2d revealed that the prevalence of liver fibrosis was significantly higher in patients with DM, hypertension, metabolic syndrome, respectively (all P < 0.05). The prevalence of liver fibrosis was higher in patients with hyperlipidemia than the control group, but the difference was not statistically significant (P > 0.05).

#### Effects of various drugs on the prevalence of liver fibrosis

Fig. 2e illustrated that the prevalence of liver fibrosis was higher in patients taking antipsychotic polypharmacy than single couplet medicine, but the difference was not statistically significant (P > 0.05). Furthermore, the prevalence of liver fibrosis was higher in patients receiving therapy for hypertension, dyslipidemia and diabetes mellitus, but the differences were not statistically significant (P > 0.05).

To explore the differences of liver fibrosis in subjects using different antipsychotic drugs, we further selected six drugs that were commonly used in these patients, including olanzapine, clozapine, risperidone, chlorpromazine, quetiapine, aripiprazole. As shown in Fig. 2f, there was no statistically significant difference in the prevalence of liver fibrosis among the 450 patients treated with each of the six antipsychotic drugs alone.

### Binary logistic regression analysis of risk factors for liver fibrosis in psychiatric patients

We took liver fibrosis as the dependent variable and conducted binary Logistic regression analysis to explore the independent risk factors of it in psychiatric patients. As shown in Table 3, we can see that age, BMI and VAI were independent risk factors for liver fibrosis in psychiatric patients. Additionally, application of more antipsychotics was suggested to be associated with an increased risk of significant fibrosis. Among patients with liver steatosis, male sex, BMI, VAI, duration of the disease and metabolic syndrome were independent risk factors for liver fibrosis. Meanwhile, application of more antipsychotics was also suggested to be associated with an increased risk of significant fibrosis. By contrast, among patients without liver steatosis, only age and BMI were independent risk factors for liver fibrosis.

**Table 3:** Binary Logistic regression analysis for liver fibrosis in psychiatric patients

	All the psychiatric patients(n = 619)		Patients with liver steatosis(n = 302)		Patients without liver steatosis (n = 317)	
Variable	OR (95%CI)	p value	OR (95%CI)	p value	OR (95%CI)	p value
Age	**1.040(1.009 ~ 1.072)**	0.012	1.020(0.980 ~ 1.062)	0.335	**1.054(1.014 ~ 1.096)**	0.007
Sex	0.605(0.337 ~ 1.088)	0.093	**0.316(0.157 ~ 0.635)**	0.001	0.939(0.398 ~ 2.216)	0.886
Drinking	0.538(0.169 ~ 1.716)	0.295	0.425(0.082 ~ 2.211)	0.309	1.125(0.229 ~ 5.517)	0.885
BMI	**1.177(1.079 ~ 1.283)**	0.000	**1.223(1.092 ~ 1.369)**	0.000	**1.153(1.015 ~ 1.309)**	0.028
VAI	**1.141(1.015 ~ 1.282)**	0.027	**1.161(1.027 ~ 1.313)**	0.017	1.079(0.776 ~ 1.499)	0.653
Duration of the disease	1.009(0.988 ~ 1.030)	0.389	**1.041(1.011 ~ 1.073)**	0.008	0.986(0.961 ~ 1.011)	0.270
Antipsychotic polypharmacy	2.054(0.946 ~ 4.461)	0.069	2.295(0.900 ~ 5.854)	0.082	0.958(0.265 ~ 3.461)	0.948
Liver steatosis	0.952(0.507 ~ 1.786)	0.877	…	…	…	…
Metabolic syndrome	1.628(0.813 ~ 3.263)	0.169	**3.162(1.090 ~ 9.169)**	0.034	0.742(0.264 ~ 2.083)	0.571

Data were odds ratio (95% confidence interval)

BMI body mass index, VAI visceral adiposity index, OR odds ratio, CI confidence interval

## Discussion

In this relatively large cross-sectional hospital-based study, a large number of patients with mental illnesses had an unsatisfactory metabolic profile compared with the general population. A significant proportion of psychiatric patients also had increased LSM. Patients with obesity and antipsychotic polypharmacy have a higher tendency towards having liver fibrosis.

We found that compared with the general population, psychiatric patients exhibited a higher prevalence of metabolic disorders including obesity, diabetes, hypertension and dyslipidemia. Our results are consistent with previous epidemiological studies [9, 10, 32–34]. Growing evidence showed that pharmacological interventions in addition to lifestyle modification could significantly reduce overall cardiovascular disease risk in adults with serious mental illness [11]. Actually, blood glucose, lipid profiles, and blood pressure were significantly lower in the recruited psychiatric patients in the two mental health care centers in Shanghai since they have easier access to health-care system resources.

To the best of our knowledge, the present study was the first to report the prevalence of liver fibrosis in patients with mental illnesses in China. The overall prevalence of liver steatosis and fibrosis is 48.7% and 15.5% in our study population, much higher than the general population based on previous reports [35, 36]. Similar to our study, a recent Australian study found that advanced liver disease, defined by LSM ≥ 8.2 kPa, was identified in 10.3% of patients with severe mental illness [37]. Older subjects, males, and those with lower education level have a higher prevalence of liver fibrosis, although not reaching statistical significance. Meanwhile, the prevalence of liver fibrosis was significantly higher in subjects with worse metabolic profile, including obesity, hypertension, diabetes and liver steatosis. Hence, metabolic-related factors may be more important in guiding liver assessment in psychiatric patients, which warrants further investigation. However, we did not observe a significant lower prevalence of liver fibrosis in the patients who were treated for diabetes, hypertension and dyslipidemia. Further well-designed randomized controlled trials should be conducted to explore whether lowering blood glucose, lipids and blood pressure could have protective effects in preventing or lowering liver fibrosis.

Although FibroScan is safe and convenient to perform, it is unlikely that clinicians could use it to all psychiatric patients because of the large number of patients and the unavailability of measurement. Hence, it is of clinical significance to identify patients at risk for advanced liver fibrosis. Traditional risk factors such as aging, general obesity and central obesity were independent factors associated with increased LSM in all psychiatric patients and in those with liver steatosis. Among psychiatric patients with liver steatosis, metabolic syndrome and disease durations were also positively associated with increased LSM. Meanwhile, among their counterparts without liver steatosis, only aging and general obesity were positively associated with increased LSM. In other words, patients with mental illnesses and obesity are at higher risk of having liver fibrosis regardless of steatosis status and may benefit from liver assessment.

Interestingly, although the prevalence of liver fibrosis was similar among subjects using different types of single antipsychotic drug, application of more antipsychotic drugs was suggested to be associated with an increased risk of significant fibrosis in all psychiatric patients and in patients with liver steatosis. There has been increasing concern that antipsychotic drugs seem to have an adverse effect on metabolic profile [38]. Growing evidence suggests that patients on atypical antipsychotics gained more weight and tend to develop diabetes and dyslipidemia after drug initiation, with clozapine and olanzapine associated with the highest metabolic risk [39]. Our study provides further evidence that antipsychotic polypharmacy may have an adverse health effect on liver fibrosis. Hence, it is important to monitor for the occurrence and progression of liver fibrosis in patients receiving antipsychotic polypharmacy, especially among those with liver steatosis.

It is worth mentioning that among psychiatric patients with BMI < 25 kg/m² and normal waist circumference, approximately 30% still had increased CAP and 9.0% had increased LSM. In previous population studies, NAFLD and advanced fibrosis are also observed in a small but significant proportion of non-obese subjects [40]. Such patients usually have other components of metabolic syndrome and in spite of relatively normal BMI often have recent weight gain [41].

Our study has the strength of a relatively large sample and the application of one of the best and widely available non-invasive tests of liver steatosis and fibrosis. In addition, to the best of our knowledge, we are the first to study the prevalence of liver fibrosis in the vulnerable group of psychiatric patients in China. There are several limitations to our study. First, we do not have a control population to compare the prevalence of liver fibrosis between the general population and psychiatric patients. Second, in our study, 85.2% of all the psychiatric patients were schizophrenic and the proportion of other types of psychosis was very small. Due to the small sample size included in this study, the risk factors for liver fibrosis in each psychosis could not be analyzed. Third, we didn’t keep detailed records on the dosage of psychiatric patients. Thus, we failed to explore whether there is dose-response association between dosage of drug use and liver fibrosis. Fourth, we used Fibroscan to measure steatosis and fibrosis. Although liver biopsy is well acknowledged as the reference standard for liver assessment, it is impractical in large population studies. Fifth, there is no universal cut-off guideline for CAP and LSM score. However, we used various cutoff points from previous Chinese studies.

## Conclusion

Psychiatric patients at hospital have a high prevalence of liver steatosis and fibrosis. Aging, obesity and antipsychotic polypharmacy are associated with liver fibrosis. Our data support coordinated and concerted effort for screening advanced liver diseases in patients with mental disorders.

## Electronic supplementary material

Below is the link to the electronic supplementary material.

Supplementary Material 1 Supplemental Table 1: Demographic characteristics of psychiatric patients

## Data Availability

The datasets during and/or analyzed during the current study are available from the corresponding author on reasonable request.

## **Abbreviations**

CAP: Controlled attenuation parameter
LSM: Liver stiffness measurement
SMI: Severe mental illness
NC: Neck circumference
WC: Waist circumference
HC: Hip circumference
BMI: Body mass index
NAFLD: Nonalcoholic fatty liver disease
HbA1c: Glycated hemoglobin
FPG: Fasting plasma glucose
TG: Triglycerides
TC: Total cholesterol
LDL: Low-density lipoprotein
LAP: Lipid accumulation product
SD: Standard deviation
CI: Confidence interval
