# Supplementary material for: High prevalence of metabolic diseases, liver steatosis and fibrosis among Chinese psychiatric patients
Source: BMC Psychiatry. 2023 Mar 28;23:206. doi: 10.1186/s12888-023-04684-1 (PMC10052799; doi:10.1186/s12888-023-04684-1)
Supplement: Supplementary file 1 — Supplementary Material 1 Supplemental Table 1: Demographic characteristics of psychiatric patients [file 12888_2023_4684_MOESM1_ESM.docx]

| **Supplemental Table 1: Demographic characteristics of psychiatric patients** | | | | | | | | | |
| --- | --- | --- | --- | --- | --- | --- | --- | --- | --- |
|  | sex | |  | educational status | |  | age (years) | |  |
|  | men | women | p value | low | high | p value | <60 | ≥60 | p value |
| Carotid plaque | 53.3% | 39.2% | 0.000 | 51.8% | 41.9% | 0.023 | 23.8% | 57.4% | 0.000 |
| Liver steatosis | 46.5% | 51.7% | 0.177 | 48.8% | 50.2% | 0.741 | 49.0% | 48.6% | 0.924 |
| Liver fibrosis | 16.7% | 14.0% | 0.336 | 16.8% | 14.0% | 0.377 | 12.9% | 16.7% | 0.209 |
| Hypertension | 54.3% | 57.0% | 0.489 | 60.4% | 50.2% | 0.020 | 39.6% | 62.3% | 0.000 |
| Hyperlipemia | 70.5% | 66.4% | 0.262 | 67.1% | 74.4% | 0.068 | 70.8% | 67.9% | 0.456 |
| Diabetes Mellitus | 31.1% | 33.9% | 0.436 | 36.9% | 31.2% | 0.170 | 24.3% | 35.8% | 0.003 |
| Central obesity | 47.3% | 84.3% | 0.000 | 63.4% | 66.0% | 0.531 | 61.9% | 63.6% | 0.673 |
| Metabolic syndrome | 39.7% | 71.0% | 0.000 | 54.9% | 57.2% | 0.593 | 49.5% | 54.6% | 0.225 |
| BMI (kg/m²) | 23.4(21.2~26.3) | 24.2(21.5~26.9) | 0.136 | 23.4(21.3~26.6) | 24.1(21.3~26.7) | 0.649 | 24.3(21.3~27.3) | 23.6(21.2~26.2) | 0.091 |
| WC (cm) | 88.5(81~95.5) | 90(83.4~96.2) | 0.163 | 89.7±10.9 | 89.4±10.3 | 0.787 | 89.1±10.5 | 89.4±10.5 | 0.772 |
| NC (cm) | 37(35~39) | 33(31.3~35) | 0.000 | 35.5(33~38) | 35(32.5~37.5) | 0.157 | 35(33~38) | 35(32.7~38) | 0.750 |
| HC (cm) | 92(88~97) | 94.5(89.5~100) | 0.002 | 93(88.5~98) | 94(89~100) | 0.064 | 94(89~100) | 93(89~98) | 0.113 |
| SBP (mm Hg) | 129(116~146) | 130(114~147) | 0.922 | 132(117~147) | 128(114~143) | 0.053 | 125(112~137) | 133(117~149) | 0.000 |
| DBP (mm Hg) | 77(69~86) | 75(66~85) | 0.049 | 77(69~87) | 77(68~84) | 0.238 | 79(71~88) | 75(67~84) | 0.000 |
| TG (mmol/L) | 1.2(0.85~1.7) | 1.3(0.92~1.9) | 0.107 | 1.26(0.88~1.71) | 1.24(0.92~1.89) | 0.607 | 1.34(0.95~2.05) | 1.21(0.85~1.66) | 0.004 |
| HDL (mmol/L) | 1.0(0.87~1.1) | 1.2(1.04~1.4) | 0.000 | 1.09(0.91~1.28) | 1.07(0.94~1.27) | 0.966 | 1.04(0.9~1.21) | 1.09(0.94~1.29) | 0.012 |
| TC (mmol/L) | 4.3±0.9 | 4.8±1.0 | 0.000 | 4.46±0.94 | 4.57±0.99 | 0.164 | 4.36(3.89~5.18) | 4.48(3.84~5.09) | 0.876 |
| LDL (mmol/L) | 2.7±0.6 | 3.1±0.7 | 0.000 | 2.81±0.69 | 2.91±0.71 | 0.110 | 2.81(2.39~3.36) | 2.81(2.4~3.29) | 0.829 |
| LAP | 28.9(14.8~49.2) | 39.4(25.2~66.7) | 0.000 | 33(19.7~57.5) | 35.1(20.6~59.7) | 0.421 | 34.4(19.8~64.5) | 32.9(18.2~55) | 0.107 |
| VAI | 1.7(1.05~2.6) | 2.3(1.43~3.5) | 0.000 | 1.81(1.21~2.81) | 1.99(1.29~2.98) | 0.239 | 2.1(1.31~3.36) | 1.8(1.16~2.8) | 0.011 |
| HbA1c (%) | 5.4(5.0~5.8) | 5.4(5.1~5.9) | 0.131 | 5.4(5.1~5.98) | 5.4(5.1~5.7) | 0.051 | 5.3(4.9~5.6) | 5.4(5.1~5.9) | 0.000 |
| FPG (mmol/L) | 5.3(4.8~6.1) | 5.5(4.9~6.2) | 0.044 | 5.41(4.9~6.2) | 5.5(4.9~6.1) | 0.768 | 5.2(4.8~5.9) | 5.45(5~6.2) | 0.001 |

Continuous variables were expressed as the mean ± standard deviation (SD) or the median with an interquartile range (25%, 75%), and categorical variables were presented as percentages (%).

LAP lipid accumulation product, VAI visceral adiposity index, BMI body mass index，WC waist circumference, NC neck circumference, HC hip circumference, SBP systolic blood pressure, DBP diastolic blood pressure, TG triglycerides, HDL high-density lipoprotein, TC total cholesterol, LDL low-density lipoprotein, FPG fasting plasma glucose, HbA1c glycated hemoglobin
